# Multipoint 5D flow cardiovascular magnetic resonance - accelerated cardiac- and respiratory-motion resolved mapping of mean and turbulent velocities

**DOI:** 10.1186/s12968-019-0549-0

**Published:** 2019-07-22

**Authors:** Jonas Walheim, Hannes Dillinger, Sebastian Kozerke

**Affiliations:** Institute for Biomedical Engineering, University and ETH Zurich, Gloriastrasse 35 8092, Zurich, Switzerland

**Keywords:** 4D flow MRI, Velocity mapping, Turbulent kinetic energy, Respiratory motion compensation, Cartesian Golden angle, Low-rank image reconstruction, Cardiovascular magnetic resonance

## Abstract

**Background:**

Volumetric quantification of mean and fluctuating velocity components of transient and turbulent flows promises a comprehensive characterization of valvular and aortic flow characteristics. Data acquisition using standard navigator-gated 4D Flow cardiovascular magnetic resonance (CMR) is time-consuming and actual scan times depend on the breathing pattern of the subject, limiting the applicability of the method in a clinical setting.

We sought to develop a 5D Flow CMR framework which combines undersampled data acquisition including multipoint velocity encoding with low-rank image reconstruction to provide cardiac- and respiratory-motion resolved assessment of velocity maps and turbulent kinetic energy in fixed scan times.

**Methods:**

Data acquisition and data-driven motion state detection was performed using an undersampled Cartesian tiny Golden angle approach. Locally low-rank (LLR) reconstruction was implemented to exploit correlations among heart phases and respiratory motion states. To ensure accurate quantification of mean and turbulent velocities, a multipoint encoding scheme with two velocity encodings per direction was incorporated. Velocity-vector fields and turbulent kinetic energy (TKE) were obtained using a Bayesian approach maximizing the posterior probability given the measured data. The scan time of 5D Flow CMR was set to 4 min.

5D Flow CMR with acceleration factors of 19 .0 ± 0.21 (mean ± std) and velocity encodings (VENC) of 0.5 m/s and 1.5 m/s per axis was compared to navigator-gated 2x SENSE accelerated 4D Flow CMR with VENC = 1.5 m/s in 9 subjects. Peak velocities and peak flow were compared and magnitude images, velocity and TKE maps were assessed.

**Results:**

While net scan time of 5D Flow CMR was 4 min independent of individual breathing patterns, the scan times of the standard 4D Flow CMR protocol varied depending on the actual navigator gating efficiency and were 17.8 ± 3.9 min on average. Velocity vector fields derived from 5D Flow CMR in the end-expiratory state agreed well with data obtained from the navigated 4D protocol (normalized root-mean-square error 8.9 ± 2.1%). On average, peak velocities assessed with 5D Flow CMR were higher than for the 4D protocol (3.1 ± 4.4%).

**Conclusions:**

Respiratory-motion resolved multipoint 5D Flow CMR allows mapping of mean and turbulent velocities in the aorta in 4 min.

## Background

The hemodynamic assessment of blood flow through heart valves and in the great vessels using 4D Flow cardiovascular magnetic resonance (CMR) has received increasing attention and a number of important applications have emerged [1]. While standard parameters including volume flow and peak velocities have been widely used, a number of additional readouts have been suggested. Among these readouts, probing the intravoxel velocity standard deviation [2] holds considerable promise as it permits estimating turbulent contributions in blood flow [3–5].

A number of clinical studies have indicated the added value of measuring turbulent velocities alongside mean velocities using 4D Flow CMR [2, 6, 7], e.g. by the assessment of turbulent kinetic energy (TKE) which indicates how much energy is stored in turbulent flow and which has shown elevated values for patients with bicuspid aortic valves or a dilated aorta relative to healthy subjects [7]. Ongoing studies promise further insights into the clinical relevance of turbulent flow estimation [8].

A key challenge of encoding mean and turbulent velocities relates to achieving appropriate measurement sensitivity to both quantities. For the expected ranges of mean and turbulent velocities in-vivo, multiple velocity encodings per spatial direction are preferably obtained and combined during image reconstruction [4]. This approach, however, requires additional data and hence data undersampling is necessary to arrive at clinically acceptable scan times.

Significant scan time reductions in 4D Flow CMR have been achieved using combinations of parallel imaging and compressed sensing [9], k-t methods [10, 11], radial [12] or spiral [13] acquisitions. Various modifications to the way data are sampled and reconstructed have been described, e.g. [14–19]. In general, however, scans remain too long to be performed in breathholds. Moreover, data acquisition without respiratory motion compensation leads to decreased image quality [14]. Accordingly, navigator-based respiratory gating is typically employed [20]. Unfortunately, respiratory gating makes scan times unpredictable, as gating efficiencies vary among subjects and often even during a single scan. Clinically, this may lead to conflicts as scan slots have typically fixed durations.

Instead of using respiratory gating, respiratory-motion resolved imaging [21–24] has been proposed to address respiratory-motion related image artifacts. To this end, data are acquired continuously throughout the respiratory motion cycle and sorted afterwards during the image reconstruction task into discrete motion states based on a respiratory motion signal surrogate [23, 25]. Accordingly, data correlation not only along the cardiac phase dimension but also along the respiratory motion dimension can be exploited.

Data correlations lead to low-rank properties of the decoding problem to be solved in image reconstruction. Various studies have demonstrated that these low-rank characteristics are most efficiently exploited using local rather than global low-rankedness using patch-based decomposition of the multi-dimensional data [26, 27].

The objective of the present work was to implement and test respiratory-motion resolved Bayesian multipoint 5D Flow CMR for mapping both mean and turbulent velocities in the aorta with a fixed scan time.

## Methods

### Data acquisition and respiratory motion binning

**Fig. 1:**
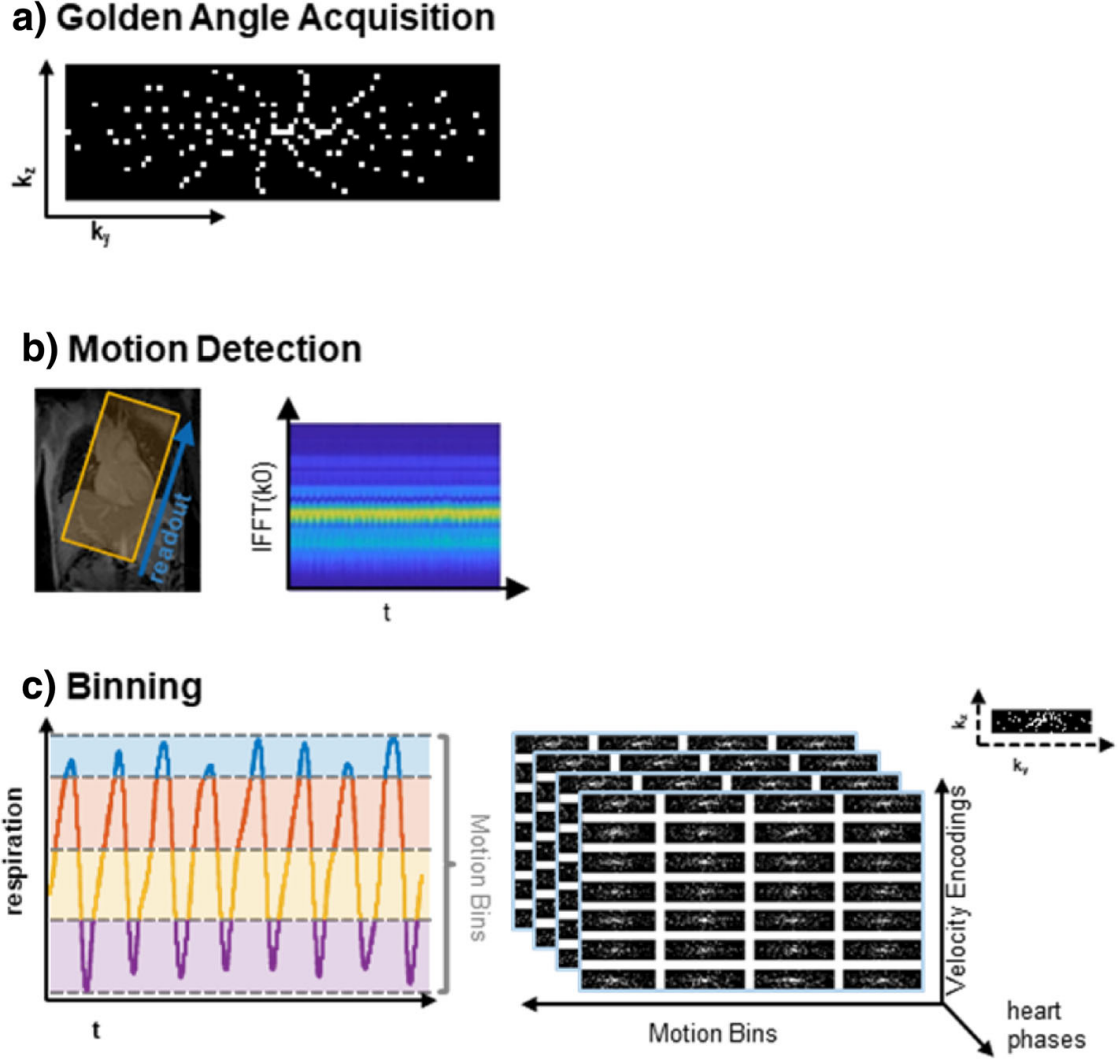
5D Flow data acquisition. **a** Data are sampled using a Cartesian pseudo-radial tiny Golden angle sampling pattern [33]. **b** Respiratory motion state detection is derived from profile d(k_x_, k_y_ = 0, k_z_ = 0), which is repeatedly acquired and processed using a combination of principal component analysis, lowpass filtering, and coil-clustering [36]. Each spoke is composed of 21 k-space profiles, one of them always through the k-space center . With *T*_*r*_ = 3.3. *ms*, respiratory motion is sampled at ca. 14 Hz. **c** Acquired data are sorted into four discrete respiratory motion states such that the acceleration factor for each motion state is similar

The data acquisition and motion binning process is illustrated in Fig. 1. Data are sampled using a pseudo-radial Cartesian sampling pattern which maps radial spokes onto a Cartesian grid [28, 29]. Using the Golden angle principle [30, 31], spatial incoherence of temporally adjacent frames is ensured. To reduce eddy current effects and keep the acoustic noise level during the measurement to a minimum, the tiny Golden angle concept was employed to avoid large jumps in k-space [31].

**Fig. 2:**
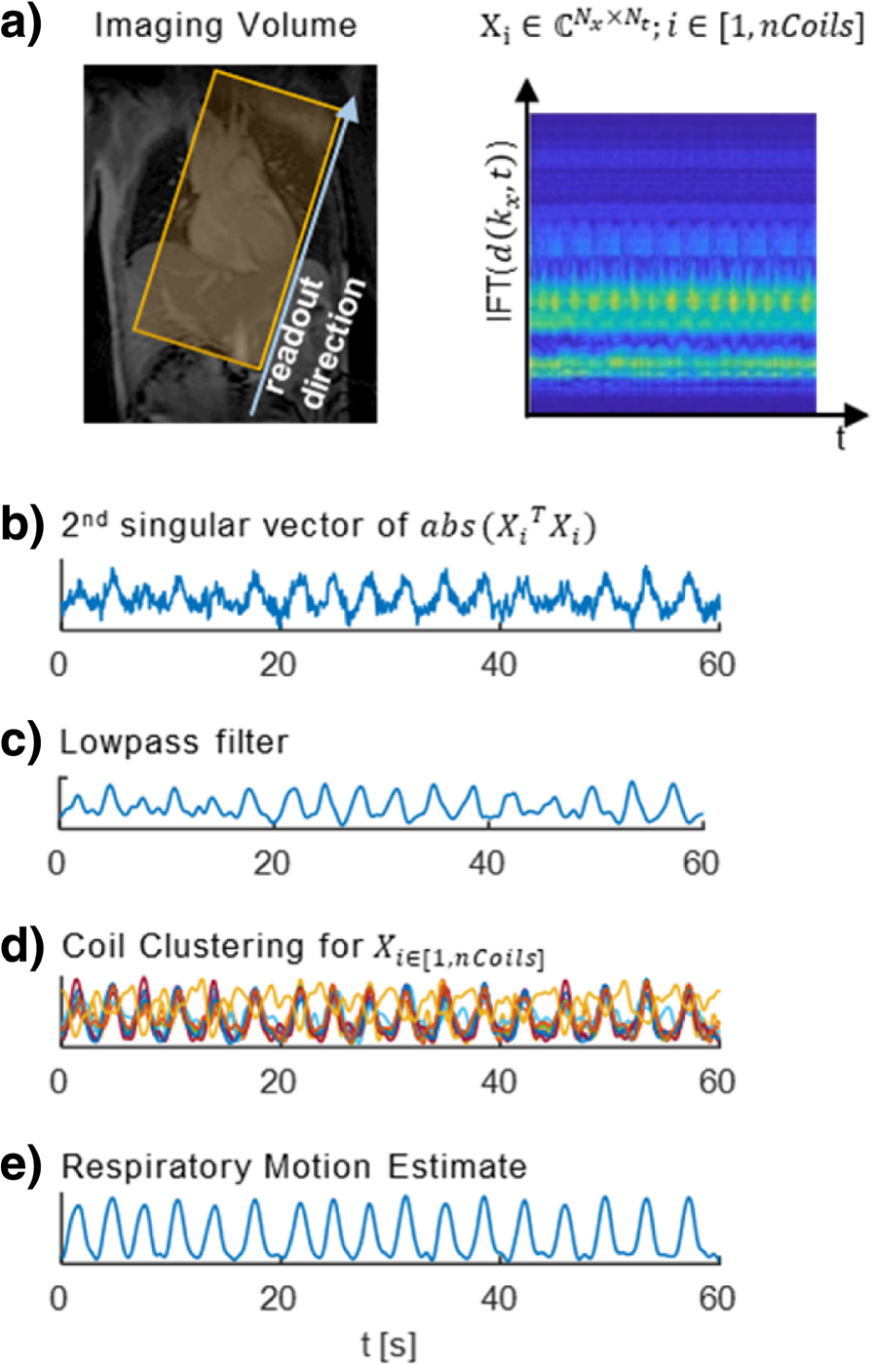
Data driven respiratory motion detection. **a** Respiratory motion is extracted from the profiles through the k-space center *d*(*k*_*x*_, *k*_*y*_ = 0, *k*_*z*_ = 0). The temporal evolution of readouts through the k-space center shows a modulation with respiratory motion and cardiac motion. **b** The 2nd principal component provides the main variation of the signal. **c** A lowpass filter limits the spectral components to the expected frequency range of respiratory motion. **d**) Coil clustering is used to combine the signals from different channels and provides a final estimate of respiratory motion

Data-driven respiratory motion detection [32] was performed as illustrated in Fig. 2a) using repetitive sampling of profiles through the k-space center *d*(*k*_*x*_, *t*) = *d*(*k*_*x*_, *k*_*y*_ = 0, *k*_*z*_ = 0, *t*) as part of the pseudo-radial Cartesian sampling pattern. Upon inverse Fourier transform of *d*(*k*_*x*_, *t*), the projection *X*(*x*, *t*) of the excited volume resolved along the frequency encode direction *x* and over time *t* was obtained, showing signal modulation depending on respiratory and cardiac motion. Given *TR* = 3.3 *ms* and with one in 21 readouts through the k-space center, a respiratory sampling rate of ca. 14 Hz was obtained. The signal of each coil channel was treated as a separate signal ${\mathrm{X}}_{\mathrm{i}}\in {\mathbb{C}}^{N_x\times {N}_t};i\in \left\{1,2,\dots, nCoils\right\}$, with *N*_*x*_ being the number of samples in readout dimension and *N*_*t*_ the number of readouts through the k-space center. Only the magnitude was considered, as the main variation in signal phase was found to be related to the different velocity encodings which were alternated per heart beat (beat-interleaved) and which led to phase variations which were too large to be eliminated by lowpass filtering.

Singular value decomposition was performed and the 2nd singular vector of abs(*X*^*T*^*X*) was selected, as it provided the predominant motion for each coil channel (Fig. 2b). To isolate respiratory motion, a lowpass filter was applied to limit the spectrum to frequencies below 0.6 *Hz* (Fig. 2c). Next, coil channel clustering [28] was performed to determine the predominant dynamics among all channels which were assumed to be the respiratory motion signal (Fig. 2d). Finally, the average over all these signals was calculated to provide the final respiratory motion signal surrogate (Fig. 2e).

### Motion-resolved image reconstruction

Using the respiratory motion surrogate signal, the acquired data were distributed across discrete motion bins. A total of four motion bins was defined and the width of each motion bin was set to achieve a similar undersampling factor for each respiratory motion state and cardiac phase by setting the bin boundaries to the 0.25-, 0.5- and 0.75-quantile of the respiratory motion signal. For some combinations of respiratory motion state, heart phase and velocity encoding, this approach led to very low sampling rates. Therefore, a data sharing approach [33, 34] was used to fill up frames with acceleration factors higher than 20 with data from neighboring respiratory motion states.

**Fig. 3:**
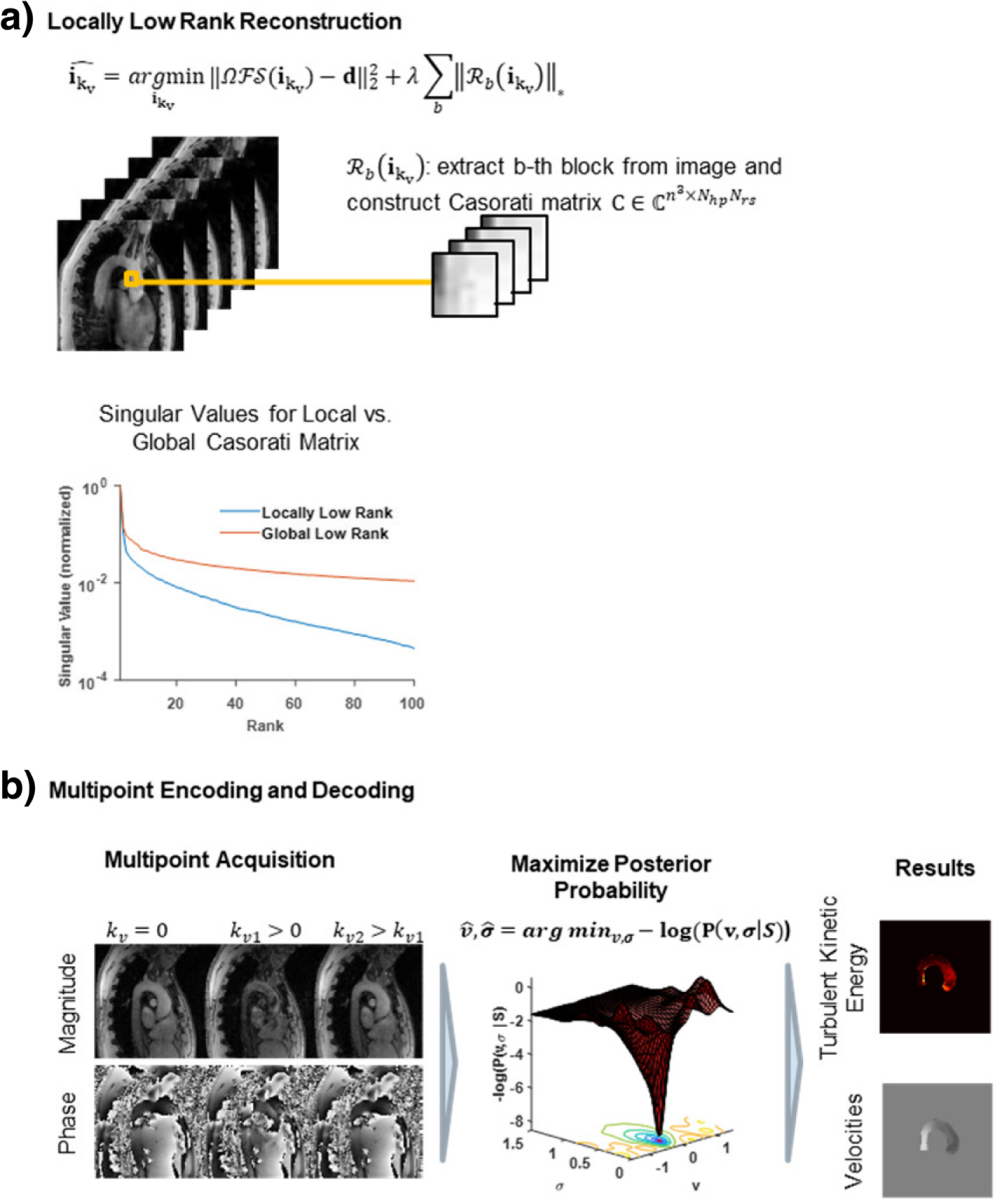
Image reconstruction using locally low rank approach followed by Bayesian multipoint unfolding. **a**) A locally low-rank approach is employed for each velocity encoding separately. The locally low-rank model divides the image into 3-dimensional patches. Each patch is reordered into local Casorati matrices for which a low rank is enforced by penalizing the nuclear norm. Compared to a global Casorati matrix, the values of the singular values decrease more rapidly. **b**) Following reconstruction of the individual velocity encodings, for each Cartesian direction the different velocity encodings *k*_*v*_ are combined using a Bayesian multipoint approach. A Bayesian probability model [4] provides posterior probabilities for mean velocity *v* and intra-voxel standard deviation *σ* given the measured signal *S*. *v* and *σ* are chosen such that the posterior probability is maximized, providing maps of turbulent kinetic energy (TKE) and mean velocities

Accordingly, the final data vector reads $\boldsymbol{d}\in {\mathbb{C}}^{N_x{N}_c{N}_{hp}{N}_{k_v}{N}_{rs}}$, with *N*_*x*_ being the number of samples in the spatial frequency domain, *N*_*c*_ denoting the number of coils, *N*_*hp*_ heart phases, ${N}_{k_v}$ velocity encodings and *N*_*rs*_ respiratory motion states. A locally-low-rank (LLR) [26, 27] approach was used to exploit correlations among heart phases (hp) and respiratory motion states (rs). Image-domain data for each velocity encoding ${\boldsymbol{i}}_{kv}\in {\mathbb{C}}^{N_x{N}_{hp}{N}_{rs}}$ were reconstructed separately as:

1 $$ {\hat{\boldsymbol{i}}}_{k_v}=\underset{{\boldsymbol{i}}_{k_v}}{\ \mathit{\arg}\min }{\left\Vert \varOmega \mathcal{FS}{\boldsymbol{i}}_{k_v}-{\boldsymbol{d}}_{k_v}\right\Vert}_2^2+\lambda {\sum}_{b=1}^{N_b}{\left\Vert\ {\mathcal{R}}_b\left({\boldsymbol{i}}_{k_v}\right)\right\Vert}_{\ast } $$

with the undersampling operator *Ω*, Fourier transform $\mathcal{F}$, coil sensitivities $\mathcal{S}$, k-space data ***d***_*kv*_ and regularization weight *λ*. As illustrated in Fig. 3a), the operator ${\mathcal{R}}_b$ selects the *b*-th out of *N*_*b*_ blocks of size *n*_*x*_ × *n*_*y*_ × *n*_*z*_ in the image from all *N*_*hp*_ heart phases and *N*_*rs*_ respiratory motion states and transforms them into a Casorati matrix with dimensions *n*_*x*_*n*_*y*_*n*_*z*_ × *N*_*hp*_*N*_*rs*_. By penalizing the nuclear norm of this local Casorati matrix, low-rankedness is enforced locally.

For comparison, respiratory-motion resolved data were also reconstructed by penalizing total variation (TV) along the respiratory motion states and heart phases [22]:

2 $$ {\hat{\boldsymbol{i}}}_{k_v}=\underset{{\boldsymbol{i}}_{k_v}}{\ \mathit{\arg}\min }{\left\Vert \varOmega \mathcal{FS}{\boldsymbol{i}}_{k_v}-{\boldsymbol{d}}_{\boldsymbol{kv}}\right\Vert}_2^2+{\lambda}_{hp}\left.T{V}_{hp}\Big({\boldsymbol{i}}_{k_v}\right)+{\lambda}_{rs}\left.T{V}_{rs}\Big({\boldsymbol{i}}_{k_v}\right) $$

where *λ*_*hp*_ and *λ*_*rs*_ denote the regularization weights along cardiac phases and respiratory motion states, respectively.

### Multipoint velocity encoding and decoding

Assuming a Gaussian distribution of intra-voxel velocities, the following signal model of mean velocity $\overline{\boldsymbol{v}}$ and fluctuating component with standard deviation ***σ*** [2] was used:

3 $$ S\left({\boldsymbol{k}}_{\boldsymbol{v}}\right)={S}_0{e}^{\frac{-{\boldsymbol{\sigma}}^2{k}_v^2}{2}}{e}^{-i{\boldsymbol{k}}_{\boldsymbol{v}}\overline{\boldsymbol{v}}} $$

where ***k***_***v***_ is related to the first gradient moment of a bipolar velocity encoding gradient by ${\boldsymbol{k}}_{\boldsymbol{v}}=\gamma {\int}_0^Tt\boldsymbol{G}(t) dt$, with *T* being the time of application of gradient waveform ***G***(*t*) and determines the encoding velocity as ${v}_{enc}=\frac{\pi }{k_v}$.

The signal model implies a trade-off between a sufficiently high encoding velocity *v*_*enc*_ to avoid phase wraps [1], and a sufficiently high encoding efficiency of the fluctuating velocity components [5] such that *v*_*enc*_~*σ*. It has been demonstrated that this trade-off may lead to insufficient sensitivity to fluctuating velocity components and hence a multipoint encoding scheme using two instead of one *k*_*v*_ encoding point per axis was implemented [4]. Using the probability map $P\left(\overline{\boldsymbol{v}},\boldsymbol{\sigma} |D,S\right)$, pixel-wise estimates of mean velocity $\overline{\boldsymbol{v}}$ and intra-voxel standard deviation ***σ*** given the model *S* and the measured image data *D* were obtained [4]. Data acquired with different ***k***_***v***_′ s were combined by choosing the values for $\overline{\boldsymbol{v}}$ and ***σ*** maximizing the posterior probability of the measured data:

4 $$ \hat{\overline{\boldsymbol{v}}},\hat{\boldsymbol{\sigma}}=\arg \underset{\overline{v},\sigma }{\max }P\left(\overline{\boldsymbol{v}},\boldsymbol{\sigma} |D,S\right). $$

An illustration of multipoint acquisition and Bayesian decoding is provided in Fig. 3b).

### In-vivo study

All in-vivo work was performed upon written informed consent of the subjects and according to local ethics regulations. Data of the ascending aorta of nine healthy subjects (30 ± 11 years, 6 male) were acquired on a 3 T CMR system (Ingenia; Philips Healthcare, Best, the Netherlands) using the proposed 5D Flow CMR and a standard, navigator-gated 4D Flow CMR approach [1] with a spatial resolution of 2.5 × 2.5 × 2.5mm^3^, *T*_*E*_ = 3.3 *ms*, *T*_*R*_ = 4.9 *ms*, flip angle = 8 and 25 cardiac phases. The different velocity encodings were alternated per heart beat. The image acquisition matrix was *N*_*readout*_ = 100 ± 9, *N*_*phase*_ = 101 ± 8 and *N*_*slice*_ = 20 ± 1 (mean ± std). Using the proposed method, velocities were encoded with *v*_*enc*_ = 50 *cm*/*s* and 150 *cm*/*s* per axis and an additional *v*_*enc*_ = 0 *cm*/*s* measurement. Scan time was fixed to 4 min. After sorting the data into 4 respiratory motion states, acceleration factors were 19.0 ± 0.21 (mean ± std). On average the view-sharing approach led to 61% of the total number of acquired samples being shared among motion bins in the study cohort.

Standard 4D Flow CMR was recorded with a single venc (*v*_*enc*_ = 150 *cm*/*s*) per axis (4D Flow Ref). Two-fold acceleration using parallel imaging [35] and standard pencil-beam navigator gating on the diaphragm (5 mm gating window) was employed with the 4D Flow reference protocol. Accordingly, the effective scan time depended on the breathing pattern of the subjects. All measurements were performed with retrospective cardiac gating and a 28-channel receiver coil.

Prior to image reconstruction, the 28-channel data were compressed to 8 virtual channels [36]. The locally low-rank (LLR) reconstruction (Equation 1) was solved using an implementation of the fast iterative shrinkage-thresholding algorithm (FISTA) [37] provided with the Berkeley advanced reconstruction toolbox (BART) [38]. A patch size of *n*_*x*_ = *n*_*y*_ = *n*_*z*_ = 8 was used for the locally low-rank constraint. The TV reconstruction (Equation 2) was performed with an adapted version of the code provided with [22]. The standard 4D Flow reference data were reconstructed with MRecon (GyroTools LLC, Zurich, Switzerland). Sensitivity maps were estimated from a separate scan using the ESPIRiT method [39].

The regularization hyperparameters *λ* in Equation 1 and *λ*_*hp*_ and *λ*_*rs*_ in Equation 2 were optimized for best agreement of velocity components in systole with data of the 4D Flow reference measurement:

5 $$ \hat{\boldsymbol{\lambda}}=\underset{\boldsymbol{\lambda}}{\mathit{\arg}\min }{\left\Vert {\boldsymbol{v}}_{recon}\left(\boldsymbol{\lambda} \right)-{\boldsymbol{v}}_{ref}\right\Vert}_1. $$

Equation 4 was minimized using the *bayesopt* function in MATLAB R2017b (The MathWorks, Natick, Massachusetts, USA) [40]. Resulting hyperparameters were *λ* = 0.01 for Equation 1 and *λ*_*hp*_ = 0.04 and *λ*_*rs*_ = 0.05 for Equation 2. For both optimizations, the number of iterations was set to 80.

### Data analysis

Upon image reconstruction, concomitant gradient terms were corrected [41] and third-order background phase correction was applied [42, 43]. Segmentation of the aorta was performed using ITK-SNAP [44]. The same masks were used for 4D Flow reference data and data in expiration obtained with 5D Flow LLR and 5D Flow TV. Maximum intensity projections (MIP) of velocity magnitude and TKE maps in systole were calculated as:

$$ {\mathit{\operatorname{Im}}}_{MIP}\left(x,y\right)=\arg \underset{z}{\max}\left(\mathit{\operatorname{Im}}\left(x,y,z\right)\right). $$

In order to avoid noise at the vessel boundaries the segmentation masks were eroded by one pixel for MIP projections.

The normalized root-mean-square error (nRMSE) of the velocity magnitude in the segmented aorta was calculated according to:

$$ nRMSE=\sqrt{\frac{\sum_{ROI}{\left(\boldsymbol{v}-{\boldsymbol{v}}_{ref}\right)}^2}{\left| ROI\right|\ast \max \left({{\boldsymbol{v}}_{ref}}^2\right)}} $$

where |*ROI*| corresponds to the number of voxels in the segmentation mask.

TKE was calculated voxel-wise as:

$$ TKE=\frac{\rho }{2}\left({\sigma}_x^2+{\sigma}_y^2+{\sigma}_z^2\right) $$

where ${\sigma}_j^2$ denotes the variance of velocity deviations per direction *j* and *ρ* is the fluid density (here we assumed 1060 *kg*/*m*^3^ for blood).

Moreover, multi-planar reslicing along the aorta was conducted using VMTK (www.vmtk.org) and peak flow and peak through-plane velocities (i.e. the maximum velocity component in the corresponding cross-section of the aorta) were assessed for each plane. Peak through-plane velocities were determined upon application of a 3x3x3 median filter to reduce contributions from noise.

The effectiveness of respiratory-motion resolved 5D Flow CMR was assessed by comparing it to reconstructions using only data from the end-expiratory bin (EXP) and to reconstructions using no respiratory binning (NG).

To assess the performance of navigator gating in the standard 4D Flow CMR protocol, the positions measured by the navigator on the diaphragm were interpolated and each readout was attributed its corresponding navigator position. Then, mean and standard deviations of the acceptance window per heart phase were calculated. Due to the coarse temporal resolution of the navigator signal (one navigator per heart beat), linear interpolation was chosen in order to provide a lower bound on the width of the acceptance window for different cardiac phases.

### Statistical analysis

Bland-Altman analysis [45] of peak flow and peak through-plane velocities was performed to assess the agreement of velocity fields obtained with 5D Flow LLR and the 4D Flow reference.

## Results

Scan durations for 4D Flow CMR were 17.8 ± 3.7 min compared to 4 min for the 5D Flow protocol.

**Fig. 4:**
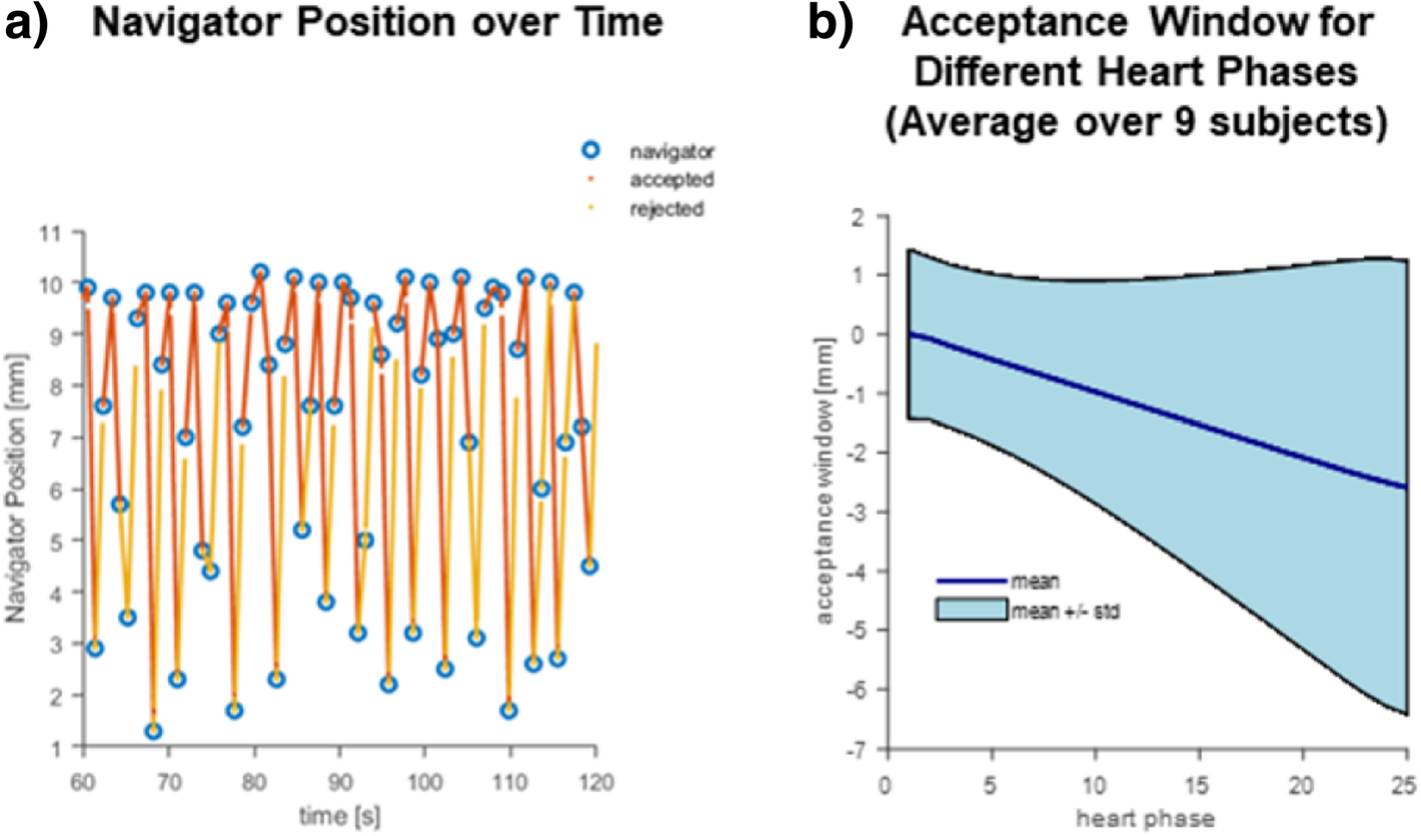
Analysis of navigator-based respiratory gating in standard 4D Flow CMR. **a**) For an exemplary 4D Flow scan the diaphragm position measured by the pencil beam navigator over time is shown together with accepted data (red) and rejected data (yellow). When the navigator signal is within the acceptance window, data of all cardiac phases are accepted until the next navigator position is obtained. As a result, the effective acceptance window of the data varies as a function of heart phase and becomes wider towards diastole as shown in **b**)

Figure 4 provides an analysis of the acceptance window for respiratory gating with one pencil beam navigator per cardiac cycle as being used in the 4D Flow reference measurements. If the navigator signal was within the acceptance window, data of all cardiac phases were accepted. This results in a wider acceptance window for later cardiac phases leading to corresponding uncertainty about the actual motion states and increasing image artifacts for later cardiac phases.

**Fig. 5:**
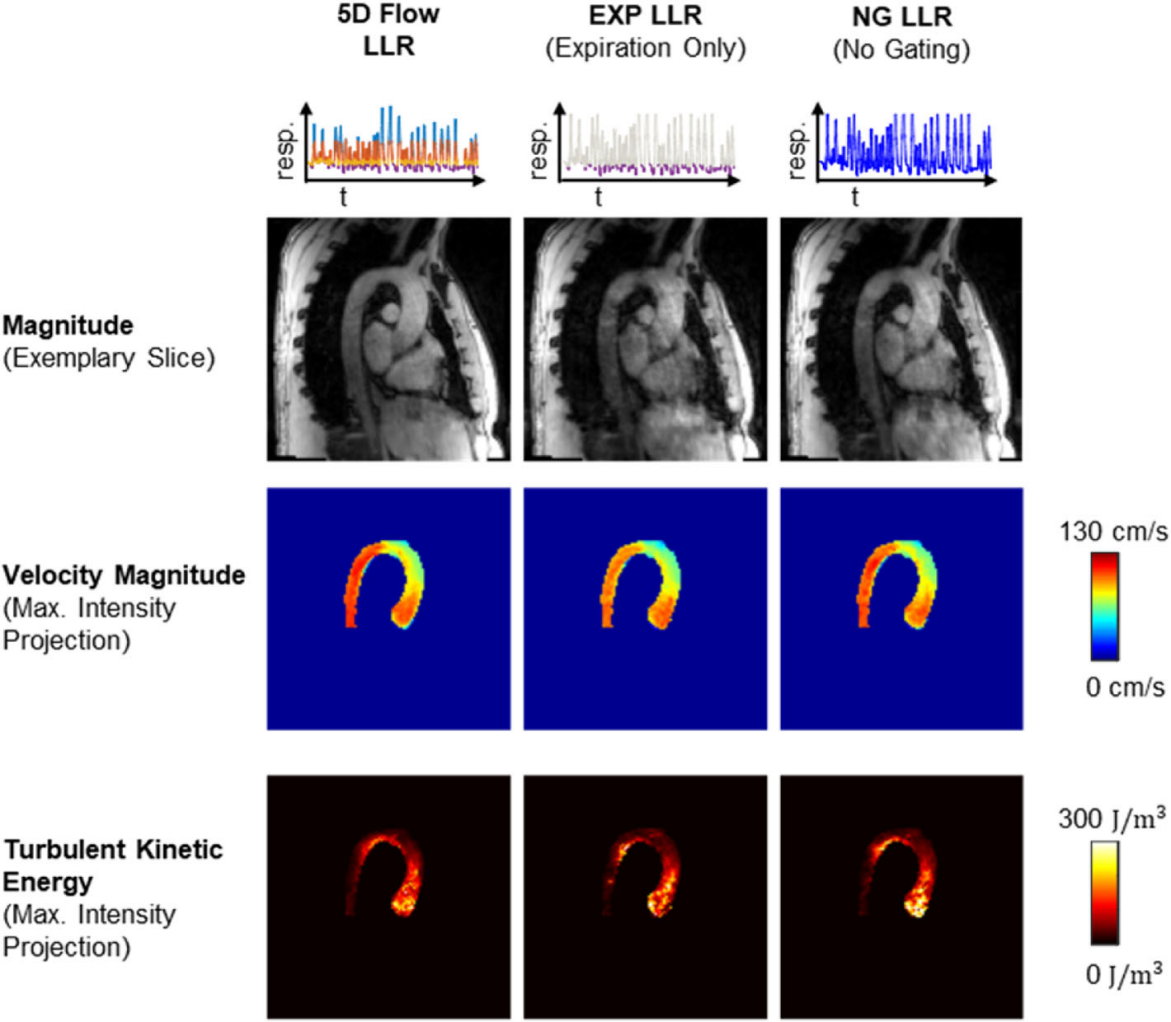
Comparison of 5D Flow LLR reconstructions in expiration relative to reconstructions using end-expiratory data only (EXP LLR) and without gating (NG LLR). 5D Flow LLR shows reduced aliasing artifacts in the magnitude, velocity and TKE maps. For both EXP LLR and NG LLR, residual motion artifacts are present in the magnitude images and increased noise is observed in the TKE maps

Reconstructions exploiting respiratory motion states (5D Flow LLR) are compared to using only data in end-expiration (EXP LLR) and without gating (NG LLR) in Fig. 5. Magnitude images and TKE maps from 5D Flow LLR reconstructions show less artifacts compared to EXP LLR and NG LLR reconstructions.

**Fig. 6:**
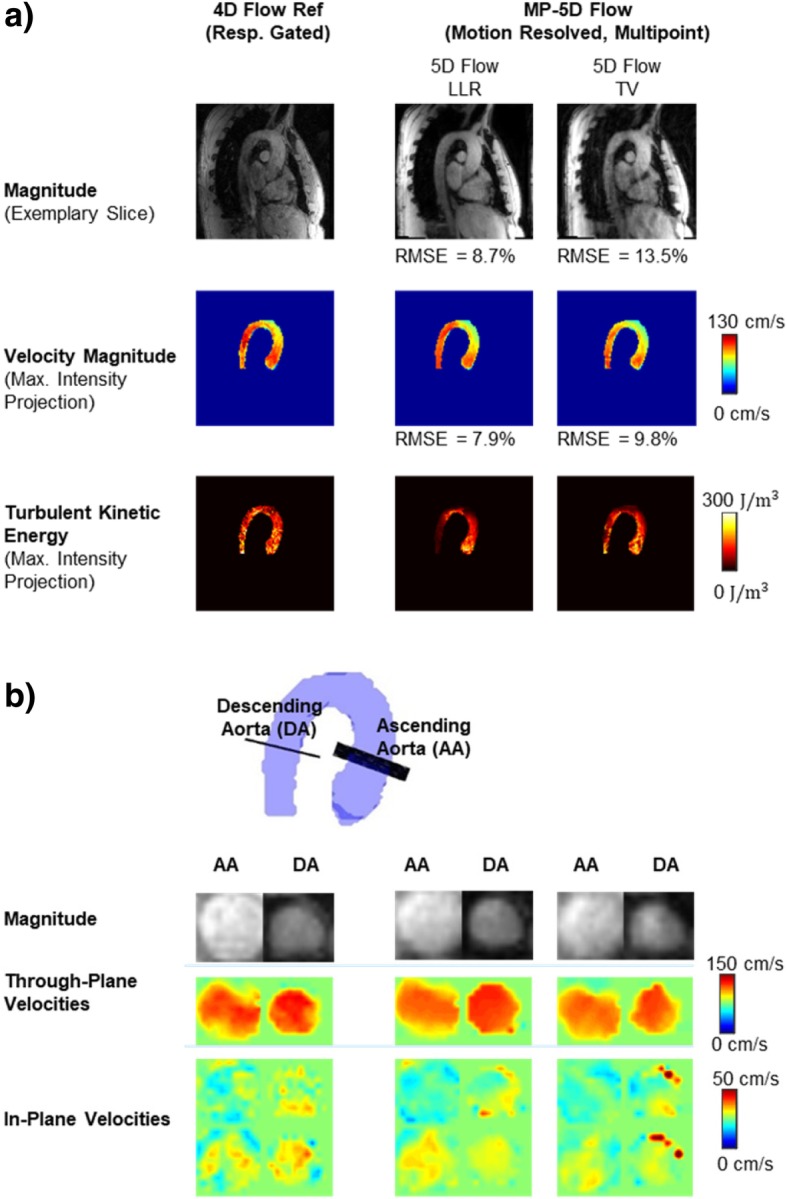
Results in systole comparing 5D Flow LLR with 5D Flow TV and standard 4D Flow. For 5D Flow TV and the 4D Flow reference residual aliasing is observed in the magnitude images. Moreover, the TKE maps obtained with 5D Flow TV and the 4D Flow reference show more noise compared to 5D Flow LLR. Exemplary slices in the ascending aorta (AA) and descending aorta (DA) show qualitatively similar results for through-plane velocities in the ascending aorta whereas in the descending aorta artifacts can be observed in the in-plane velocity components for 5D Flow TV

**Fig. 7:**
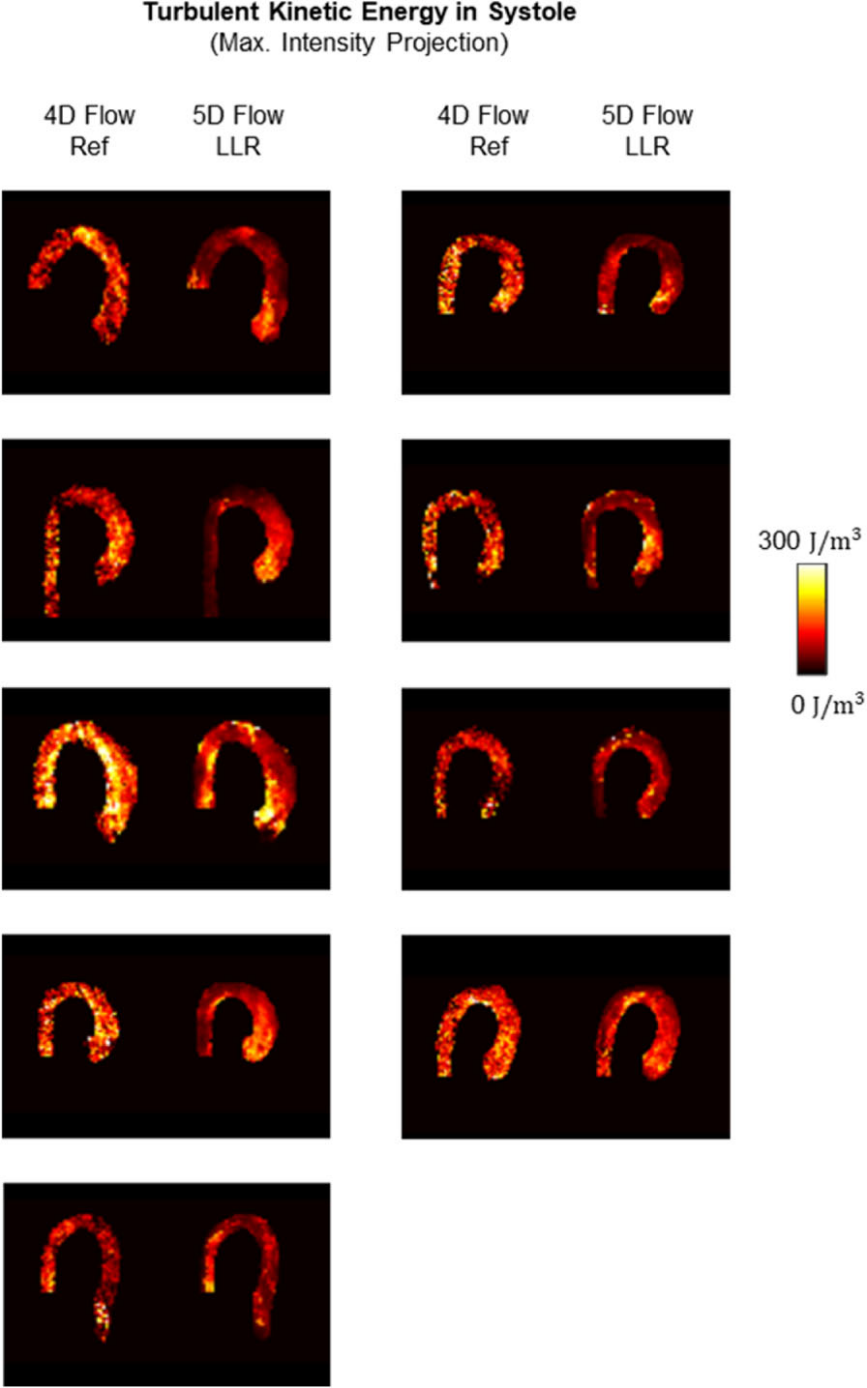
Comparison of TKE maps in systole for the 4D Flow reference versus 5D Flow LLR. In the 4D Flow reference data increased noise compared to 5D Flow LLR is seen. Moreover, TKE maps from 4D Flow show high values in the descending aorta whereas TKE values are lower in the descending aorta for most cases with 5D Flow LLR

Magnitude and velocity magnitude images for the end-expiratory motion state obtained with 5D Flow LLR and 5D Flow TV are compared relative to the 4D Flow reference in Fig. 6. It can be seen that maximum intensity projections of velocity magnitudes are similar for all methods while TKE maps and magnitude images provided by 5D Flow TV show more residual aliasing when compared to 5D Flow LLR. The root mean square estimate (RMSE) of the reconstructed images was lower for 5D Flow LLR compared to 5D Flow TV for both magnitude and velocity magnitude (8.7 vs. 13.5% for and 7.9 vs 9.8%, respectively). Maps of TKE for all 9 subjects obtained with 5D Flow LLR and the 4D Flow reference are compared in Fig. 7. In general, TKE maps derived from the 4D Flow reference show a higher noise level compared to 5D Flow data.

Exemplary slices in the ascending and descending aorta show through-plane velocity profiles to be in agreement among the 4D Flow reference and 5D Flow LLR whereas 5D Flow TV shows artifacts in the descending aorta.

**Fig. 8:**
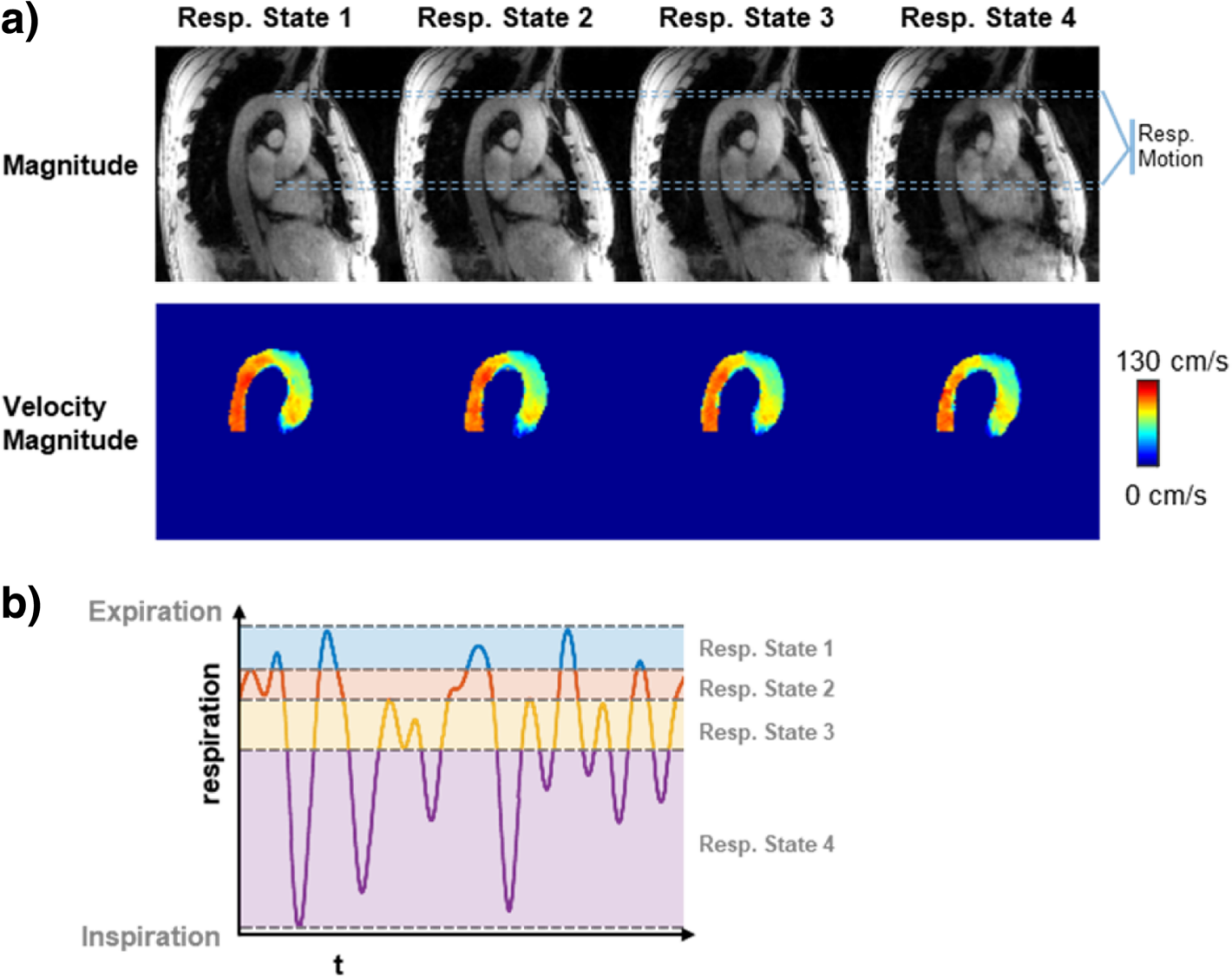
**a**) Exemplary magnitude and velocity magnitude images for different respiratory bins reconstructed with 5D LLR. Image quality reduces from end expiration to end inspiration and artifacts can be observed in the descending aorta in the magnitude and velocity images reconstructed in end inspiration. **b**): The respiratory curve shows that the expiratory bins have a narrower range of motion than inspiratory bins

Exemplary magnitude and velocity magnitude images for different respiratory bins reconstructed with 5D LLR are shown in Fig. 8 along with an exemplary respiratory curve. Bins in expiration are narrower as the duration of expiration was longer compared to inspiration. The displacement of the heart and aorta is clearly seen in both magnitude and velocity maps. While image quality is acceptable, there is degradation present from end expiration to end inspiration.

**Fig. 9:**
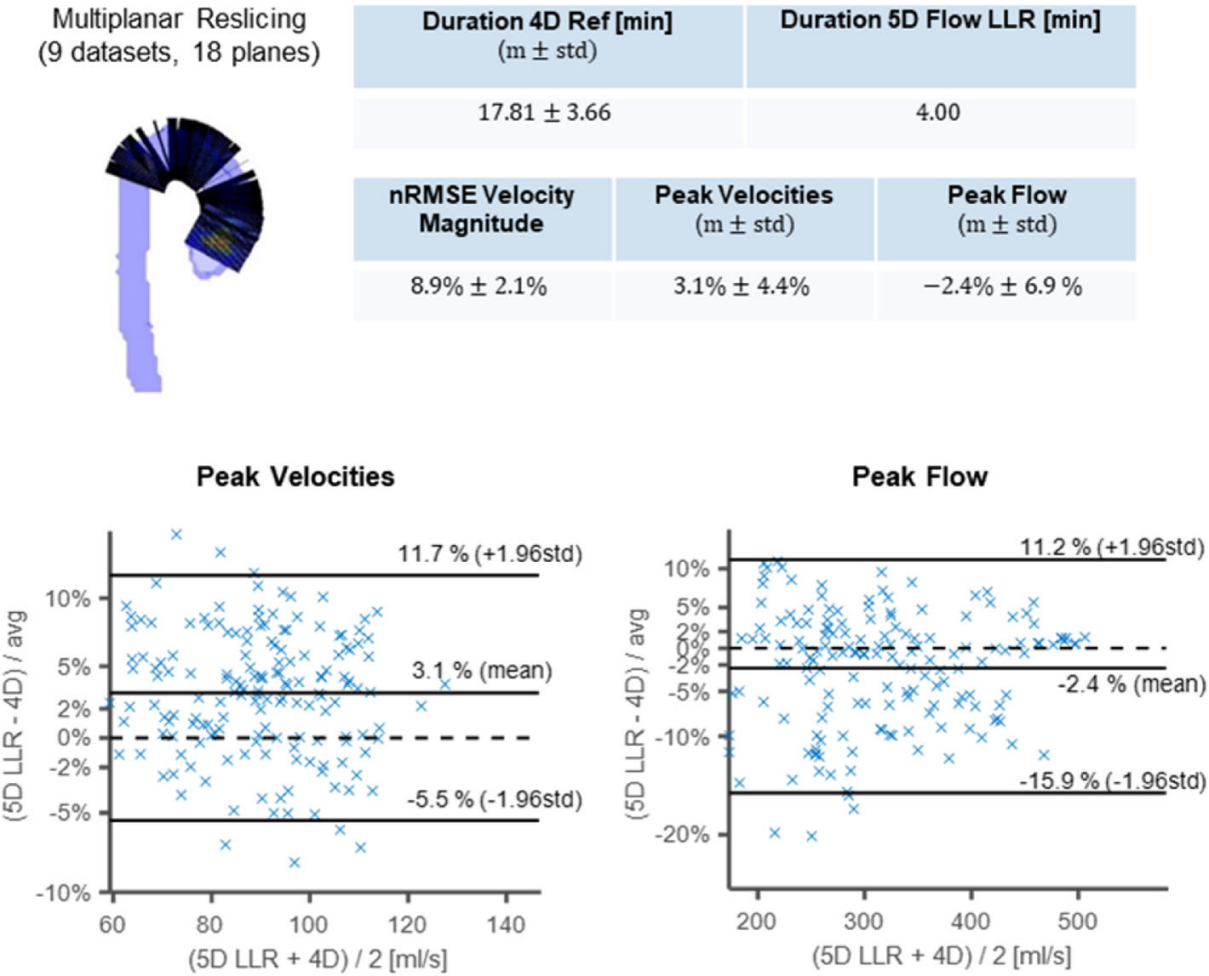
Quantitative analysis of results. Scan times, peak through-plane velocities and peak flow for 5D Flow LLR reconstructions are compared to the 4D Flow reference. Duration of the 5D Flow LLR acquisition is on average ca. 4.5 times lower than for 4D Ref. Bland-Altman analysis shows a mean difference of 3.1% and − 2.4% and limits of agreement of ±8.6% and ±13.6%

Figure 9 compares velocity fields, peak velocities and peak flow obtained for all 9 subjects. The nRMSE between the velocity magnitudes obtained with 5D Flow LLR and the 4D Flow reference was 8.9 ± 2.1%. On average, 5D Flow LLR shows good agreement of peak velocities and peak flow relative to the 4D Flow reference (peak velocities: 3.1 ± 4.4%, peak flow: −2.4 ± 6.9%).

## Discussion

In this work, a respiratory-motion resolved Bayesian multipoint 5D Flow CMR approach has been implemented based on pseudo-radial tiny Golden angle Cartesian sampling in conjunction with locally low-rank image reconstruction to map mean and turbulent velocities in the aorta in a fixed scan time of 4 min.

By exploiting data from all respiratory motion states, the duration of 5D Flow CMR becomes independent of the individual respiratory motion patterns of the subjects. In comparison to standard 4D Flow CMR protocols [1], 5D Flow CMR is about 4.5 times shorter on average. Whereas pencil-beam based navigator gating in standard 4D Flow CMR cannot ensure motion-free data across the entire cardiac cycle, as demonstrated in Fig. 4, repetitive data-driven respiratory motion detection and continuous data acquisition of 5D Flow CMR provide motion estimates throughout the cardiac cycle.

It has been demonstrated that the LLR model allows to improve reconstruction accuracy relative to using data from end expiration only, as shown in Fig. 5. Of note, low-rank models have been used to exploit correlations in dynamic CMR [46–48] and have already been proposed for reconstructing respiratory-motion resolved data. However, existing approaches confine the reconstruction to a subspace defined by basis functions along the respiratory motion dimension either by combining cardiac and respiratory motion in a Casorati matrix [21] or by means of low-rank tensor factorization [49, 50]. These approaches either require the acquisition of a separate scan to estimate a subspace or a subspace has to be estimated from undersampled k-space data, e.g. via low-rank tensor factorization [49]. The first option increases scan time and can lead to problems when there is a mismatch between both scans (e.g. bulk motion) while the second option adds complexity to the reconstruction as another hyperparameter needs to be optimized for subspace estimation prior to reconstruction of the actual image. In comparison, the LLR model proposed in the present work can be applied straight-forwardly by penalizing the nuclear norm of the data rearranged in a Casorati matrix.

When comparing the 5D Flow LLR reconstruction with the 5D Flow TV approach, both methods showed good agreement of velocity data relative to the 4D Flow reference. However, TKE maps and magnitude images exhibit less noise when using the 5D Flow LLR approach. The inferior performance of the TV approach is associated with the piecewise constant solutions favored by TV which in turn leads to smoothing of peak values. To reduce underestimation of peak velocities, hyperparameters may be determined for best agreement with reference velocities. However, this led to non-optimal results for magnitude images which therefore still showed a high degree of aliasing noise.

In-vivo, good agreement of peak velocities and peak flow values of 5D Flow LLR with data derived from standard 4D Flow CMR was found. A small bias towards higher velocities was observed for 5D Flow LLR compared to the 4D Flow reference. This can be related to previous findings that respiratory motion leads to blurring of the image [20, 51] which corresponds to spatial low-pass filtering and is therefore likely to reduce peak velocities.

A multipoint encoding scheme [4] was incorporated into the 5D Flow approach. Thereby the velocity vector field was encoded with different sensitivities to mean and fluctuating velocities to provide an accurate assessment of mean and turbulent velocities over a large dynamic range. The 7-point 5D flow LLR implementation yielded TKE maps as they can be expected for healthy, young volunteers, with moderate values of up to 300 J/m^2^ and turbulence mainly occurring in the region of the flow jet in the proximal aorta. In comparison, TKE maps derived from the 4D Flow reference with 4-point encoding appeared noisy, due to only using a single encoding velocity of 150 cm/s. Moreover, the 4D Flow reference showed TKE values in the descending aorta which were as high as in the ascending aorta. This is considered unrealistic as transient/turbulent flow typically occurs in the region downstream of the aortic valve and not in the descending aorta in healthy, young volunteers.

The acquisition time for 7-point encoded 5D Flow was fixed to 4 min. For 10-point encoding, as it has been used when assessing stenotic valves [7], this would correspond to an acquisition time of ca. 6 min for 5D Flow, compared to 17.2 ± 4.7 min for accelerated 10-point 4D Flow acquisitions [7].

Image quality of data in inspiration as shown in Fig. 8 was lower compared to data reconstructed in the end-expiratory motion state with 5D Flow LLR. This can be explained with inspiration taking a smaller fraction of the duration of the respiratory cycle than expiration. Moreover, the respiratory motion curve does not reach the maximum value in inspiration for every respiratory cycle, whereas it reaches an end-expiratory plateau in most cases. As respiratory motion states were defined to obtain similar acceleration factors in each motion state, this led to a wider range of motion in inspiratory states than in expiratory states.

The present work assessed mean and fluctuating velocity vector fields in healthy subjects only. When applying the proposed 5D Flow method in patients with aortic stenosis, an extension to 10-point encoding [7] needs to be implemented for sufficient dynamic range. Adding 3 more points would lead to an increase in scan time by ca. 42% and can thus still easily be integrated into clinical workflows.

While the present study was focused on turbulence encoding, the proposed technique can be readily applied for the acquisition of standard 4D flow CMR data in the aorta and in the heart. For different applications the scan time will change as a function of the size of the acquisition matrix, but respiratory motion is still expected to be accurately modelled as a low-rank problem. Therefore, fixing scan time independently of respiratory motion should still be feasible. Moreover, the suggested multipoint encoding approach provides improved accuracy for lower velocities, similar to other multi-VENC approaches [52, 53]. In addition, the protocol can be used to measure flow in different respiratory motion states to assess respiration dependent flow-patterns, e.g. the Fontan circulation [54, 55]. To obtain better results in inspiration, one might define bins with equal range of motion, thus leading to varying acceleration factors for different bins. In order to still obtain sufficient sampling rates in each motion state, one would therefore need to increase scan time beyond the current 4 min limit.

A limitation of the present study is the lack of a ground truth data. The 4D Flow reference data were acquired in a separate scan and consistency of flow conditions cannot be expected over the entire scan session. In addition, the 4D Flow reference data showed considerable respiratory-motion related artifacts in later cardiac phases since the pencil-beam navigator was played out right after R-wave detection and only once per cardiac cycle (acceptance rates varied between 42 and 72%). Accordingly, the quantitative comparisons with the 4D Flow data were limited to systole in order to avoid respiratory-motion corrupted cardiac phases as much as possible.

Finally, a drawback of both 5D Flow LLR and 5D Flow TV are considerably longer reconstruction times when compared to standard 4D Flow CMR (ca. 35 min for 5D LLR and ca. 60 min for 5D TV vs. ca. 3 min for conventional 4D Flow CMR on a workstation with two 14 Core Intel Xeon E5–2680 CPUs and 256 GB RAM). In the future, this relative disadvantage may be addressed by employing variational neural network based reconstructions and their deployment on dedicated hardware [56, 57].

## Conclusion

Respiratory motion resolved multipoint 5D Flow CMR allows for breathing-pattern independent mapping of mean and turbulent velocities in 4 min. The reduction in scan time allows for integration of the sequence into standard clinical workflows. Further in-vivo studies are now warranted to assess the performance of the method in relevant patient populations.

## Data Availability

The data that support the findings of this study are available from the corresponding author upon reasonable request subject to restriction on use by the Ethics Committee of the Canton of Zurich. A demo script with exemplary data will be provided online upon acceptance of this manuscript.

## Abbreviations

CMR: Cardiovascular magnetic resonance
CS: Compressed Sensing
EXP: end-expiratory
FISTA: fast iterative shrinkage thresholding
LLR: Locally-low-rank
MIP: Maximum Intensity Projection
MP: Multipoint
NG: non-gated
nRMSE: normalized root-mean-square error
RMSE: root mean square error
TKE: Turbulent kinetic energy
TV: Total Variation
VENC: Velocity encoding
